# Social Determinants of Smoking in Low- and Middle-Income Countries: Results from the World Health Survey

**DOI:** 10.1371/journal.pone.0020331

**Published:** 2011-05-31

**Authors:** Ahmad Reza Hosseinpoor, Lucy Anne Parker, Edouard Tursan d'Espaignet, Somnath Chatterji

**Affiliations:** 1 Department of Health Statistics and Informatics, World Health Organization, Geneva, Switzerland; 2 Department of Public Health, Universidad Miguel Hernández, Alicante, Spain; 3 CIBER Epidemiología y Salud Pública, Barcelona, Spain; 4 Tobacco Free Initiative, World Health Organization, Geneva, Switzerland; Genentech Inc., United States of America

## Abstract

**Introduction:**

Tobacco smoking is a leading cause of premature death and disability, and over 80% of the world's smokers live in low- or middle-income countries. The objective of this study is to assess demographic and socioeconomic determinants of current smoking in low- and middle-income countries.

**Methods:**

We used data, from the World Health Survey in 48 low-income and middle-income countries, to explore the impact of demographic and socioeconomic factors on the current smoking status of respondents. The data from these surveys provided information on 213,807 respondents aged 18 years or above that were divided into 4 pooled datasets according to their sex and country income group. The overall proportion of current smokers, as well as the proportion by each relevant demographic and socioeconomic determinant, was calculated within each of the pooled datasets, and multivariable logistic regression was used to assess the association between current smoking and these determinants.

**Results:**

The odds of smoking were not equal in all demographic or socioeconomic groups. Some factors were fairly stable across the four datasets studied: for example, individuals were more likely to smoke if they had little or no education, regardless of if they were male or female, or lived in a low or a middle income country. Nevertheless, other factors, notably age and wealth, showed a differential effect on smoking by sex or country income level. While women in the low-income country group were twice as likely to smoke if they were in the lowest wealth quintile compared with the highest, the association was absent in the middle-income country group.

**Conclusion:**

Information on how smoking is distributed among low- or middle-income countries will allow policy makers to tailor future policies, and target the most vulnerable populations.

## Introduction

Tobacco use is a leading cause of premature death and disability. Every year between five and six million deaths worldwide are attributed to tobacco use and exposure to second hand smoke [1], [2]. Over five million of these deaths are attributed directly to smoking, and about 600,000 to second hand smoke [3] i.e., people who themselves do not smoke but breathe air polluted by poisonous gases from those who smoke.

During the twentieth century, tobacco use rose to epidemic proportions, mostly due to aggressive marketing by the tobacco industry. Tobacco consumption is still rising globally, largely because the industry is targeting young people and women in many low- and middle-income countries [4]–[7]. In response to the growing epidemic, the World Health Organization sponsored the WHO Framework Convention on Tobacco Control (WHO FCTC) that calls on Parties to develop scientifically-based research evidence to assist in tobacco control efforts. Improving the global knowledge base is an important step to understand the epidemiology of smoking and in the planning, implementation and evaluation of appropriate interventions targeted at vulnerable populations.

For many low-income countries, data collected over the past decade through the Global Tobacco Surveillance System [8], the WHO STEPS program [9] and the WHO World Health Surveys (WHS) [10] have formed the mainstay of tobacco surveillance activities. Limited but very useful information has been drawn from these surveys. For example the data have shown that tobacco smoking is not equally distributed among or within countries. The highest smoking rates are seen in Europe (particularly Eastern Europe) and the Western Pacific region, while the lowest rates are seen in Africa [11]. In general, men smoke more than women both in overall consumption and in prevalence [5], although in a few countries women smoke as much as men. Examples of these countries include Brazil, Denmark and Norway [11]. In 2006, globally, an estimated 41% of men over the age of 15 smoked compared with almost nine percent of women [12]. Furthermore, smoking rates are often highest among those with the lowest levels of education and among the lowest income groups [13]–[16]. In the poorest populations, purchasing tobacco uses already scarce resources and may have important indirect effects on health. Poor rural households in china are reported to spend over 10% of their total household expenditures on cigarettes [17].

Whilst tobacco surveillance activities provide useful information for the implementation of tobacco control efforts, the World Health Surveys provide the opportunity to examine complex patterns of tobacco use and to identify the social determinants of smoking. The objective of this study is to assess demographic and socioeconomic determinants of current smoking in low- and middle-income countries. While there are some examples of studies exploring the determinants of smoking at national level [14], [15], [17], [18], there are few large cross-national datasets that use a common measurement system for smoking, as well as demographic and socioeconomic information. Such a cross-national exploration of the demographic and socioeconomic determinants of smoking can provide useful information for policy decisions, especially when focusing on low- and middle-income countries, where there is a gap in knowledge caused by limited information systems.

## Methods

### Ethics statement

Face-to-face interviews were used in all 48 countries. Written consent was obtained in all surveys. A standard consent form approved by the ethics review committee was read to the respondent in the respondent's language. Once the respondent agreed to participate in the survey, if the respondent was literate the form was provided to the respondent to read over and sign and was countersigned by the interviewer. If the respondent was illiterate and gave consent to participate, the interviewer confirmed this consent and signed on the form that the respondent had been read the form, had understood the study and agreed to participate. This procedure was approved by the institutional review boards. The full list of collaborating partners in the 48 countries where the ethical procedure is reviewed and approved is provided in List S1.

### Study population

The World Health Survey was conducted by the World Health Organization in 2002–04 to provide valid, reliable, representative and comparable population data on the health status of adults, aged 18 years and older, in 70 countries from all regions of the world [19]. All samples were probabilistically selected with every individual being assigned a known non-zero probability of being selected. The samples were nationally representative except in China, Comoros, Congo, Côte d'Ivoire, India, and the Russian Federation, where the survey was carried out in geographically limited regions. The response rates were reported in two steps: household level and individual level. The response rates at the household level were over 70% in all 48 countries except for Congo (63.6%), Swaziland (53.8%) and Czech Republic (23.9%). Individual level response rates varied between 82.2 and 100% for each country [10]. To adjust for the population distribution represented by the UN Statistical Division (http://unstats.un.org/unsd/default.htm) and also non-response, post-stratification corrections were made to sampling weights [20].

There were only a couple of high-income countries where the data for smoking were gathered in the World Health Survey, hence the setting of this study was 48 low- and middle-income countries, in which data for current smoking were available, as well as the demographic and socioeconomic factors studied. Initially 234,548 respondents were eligible but 20,741 (9%) were excluded from the analyses due to missing data on one or more variables of interest. Table S1 shows the study population and final sample size by country.

### Data

The data from 213,807 respondents aged 18 years or above were divided into 4 pooled datasets according to their sex and country income group. Country income group refers to the World Bank's development categories as of 2005 which are based on the per capita Gross National Incomes (GNI) from 2003 (the year the survey was carried out) [21]. Countries from upper and lower middle-income groups were combined for this analysis.

Current smoking was defined as a binary variable indicating whether the respondent currently smoked any tobacco product such as cigarettes, cigars or pipes. Current smokers included both daily and occasional smokers. In four countries (India, Bangladesh, Nepal and Myanmar) data were also collected on the use of smokeless tobacco. For the purpose of this analysis, individuals who only used smokeless tobacco were considered non-smokers.

The following demographic and socioeconomic factors were also included: age, (in 6 age groups: 18–29, 30–39, 40–49, 50–59, 60–69 and 70+ years), sex, marital status (in 3 groups: married or cohabiting; never married and divorced, separated or widowed), highest attained educational level (in 5 groups: no education, less than primary, primary completed, secondary or high school completed and college completed or above), whether the respondent was employed, whether the respondent lived in a rural or an urban area, and country of residence. Furthermore, a binary variable showing whether the respondent was the main economic provider for the household was also included. To evaluate wealth, a dichotomous hierarchical ordered probit model was used to develop an index of the long-running economic status of households based on owning selected assets and/or using certain service [22]. The index was divided into five quintiles within each country, where quintile 1 represents the poorest wealth quintile and quintile 5, the richest.

### Methods of analysis

The overall proportion of current smokers, as well as the proportion by each relevant demographic and socioeconomic determinant, was calculated within each of the pooled datasets. Multivariable logistic regression was used to assess the association between current smoking and the potential demographic and socioeconomic determinants according to sex and country income group. All analyses were weighted accounting for the individual survey sample designs. Specifically, each respondent in the country datasets was given a post-stratification sampling weight. This weight reflected each country's population, in such a way that if the sample size for two given countries are the same (but the population sizes of the countries are different), more weight is given to the country with higher population when calculating the pooled estimates. Stata11 was used in all analyses.

## Results

125,416 men and women from 27 middle-income countries, and 88,391 from 21 low-income countries were included in the analyses. The crude weighted prevalence of smoking was higher in the middle-income country group compared with the low-income country group for both men and for women. While this difference was only marginal in men (over 35% of men smoked in both country income groups), the prevalence of smoking in women in the middle-income country group was over double that of the low-income country group (13% compared with 6%, table 1). The crude prevalence of current smoking in each of the countries included in the analysis can be found in Table S2.

**Table 1:** Weighted average prevalence of current smoking by sex and country income group according to individual demographic and socioeconomic factors (data from the 2002–04 World Health Surveys of 48 low- or middle-income countries).

	Middle income*						Low income*					
	Male			Female			Male			Female		
	%	(95%CI	)	%	(95%CI	)	%	(95%CI	)	%	(95%CI	)
**Overall**	**40.7**	**(39.5–**	**41.8)**	**13.2**	**(12.5–**	**13.9)**	**36.1**	**(34.7–**	**37.5)**	**6.2**	**(5.5–**	**6.8)**
**Age**												
18–29	37.1	(35.4–	38.7)	12.1	(11.1–	13.2)	25.7	(23.9–	27.4)	1.9	(1.5–	2.4)
30–39	43.6	(41.5–	45.7)	15.2	(13.9–	16.5)	42.0	(39.7–	44.3)	5.3	(4.5–	6.1)
40–49	47.3	(45.2–	49.3)	16.7	(15.2–	18.3)	47.7	(45.3–	50.1)	8.7	(6.8–	10.6)
50–59	42.2	(39.9–	44.5)	14.1	(12.6–	15.7)	43.4	(40.5–	46.3)	12.8	(10.5–	15.2)
60–69	39.0	(36.2–	41.8)	10.0	(8.5–	11.5)	40.4	(36.9–	44.0)	11.7	(9.6–	13.9)
70+	27.2	(24.1–	30.3)	6.2	(5.0–	7.5)	37.8	(33.0–	42.5)	13.2	(9.9–	16.4)
**Marital Status**												
Never married	36.1	(34.4–	37.8)	13.3	(12.0–	14.6)	23.4	(21.6–	25.2)	1.9	(1.2–	2.6)
Married/cohabiting	41.8	(40.5–	43.1)	12.6	(11.8–	13.4)	41.2	(39.6–	42.8)	6.3	(5.6–	7.1)
Divorced/separated/widowed	50.5	(46.4–	54.6)	14.8	(13.4–	16.2)	40.6	(35.5–	45.7)	10.1	(8.6–	11.5)
**Education**												
No education	42.0	(38.5–	45.4)	11.4	(9.2–	13.6)	43.2	(40.8–	45.7)	8.8	(7.9–	9.8)
Less than primary	41.2	(38.0–	44.4)	18.1	(15.7–	20.5)	42.7	(40.0–	45.5)	6.0	(4.9–	7.1)
Primary completed	41.7	(39.5–	44.0)	14.2	(12.7–	15.7)	35.0	(32.9–	37.1)	3.3	(2.5–	4.1)
Secondary/high school completed	39.8	(38.4–	41.2)	12.5	(11.7–	13.3)	30.2	(28.1–	32.2)	2.0	(1.0–	3.0)
College completed/higher	41.7	(38.8–	44.7)	12.0	(10.3–	13.7)	21.2	(17.3–	25.2)	0.9	(0.4–	1.3)
**Employment**												
Not working for pay	36.8	(35.0–	38.5)	11.8	(11.0–	12.5)	22.3	(20.3–	24.2)	6.1	(5.2–	6.9)
Employed	42.3	(41.0–	43.6)	15.6	(14.5–	16.7)	39.3	(37.7–	40.8)	6.4	(5.6–	7.2)
**Main economic provider of household**												
No	37.7	(35.9–	39.4)	13.0	(12.2–	13.7)	28.4	(26.7–	30.1)	6.0	(5.3–	6.6)
Yes	42.3	(41.0–	43.5)	14.0	(12.8–	15.2)	42.2	(40.5–	43.9)	7.7	(6.4–	9.0)
**Wealth**												
Quintile 1	46.2	(44.1–	48.4)	12.9	(11.4–	14.4)	46.2	(43.4–	49.0)	8.9	(7.0–	10.9)
Quintile 2	41.9	(39.7–	44.1)	12.4	(11.2–	13.6)	41.8	(39.4–	44.3)	7.2	(6.1–	8.3)
Quintile 3	41.1	(39.0–	43.2)	13.0	(11.7–	14.2)	36.3	(33.8–	38.9)	6.9	(5.7–	8.1)
Quintile 4	39.4	(37.2–	41.6)	13.6	(12.3–	14.9)	31.3	(28.9–	33.6)	4.9	(3.9–	5.9)
Quintile 5	36.2	(34.2–	38.2)	13.8	(12.5–	15.2)	27.3	(25.1–	29.5)	3.0	(2.3–	3.7)
**Place of residence**												
Rural	42.8	(41.0–	44.6)	9.5	(8.5–	10.5)	37.3	(35.6–	39.0)	7.0	(6.2–	7.8)
Urban	39.7	(38.3–	41.1)	14.7	(13.9–	15.6)	32.5	(29.9–	35.0)	3.8	(3.0–	4.5)

*World Development Report 2005.

The prevalence of smoking was not equal in all demographic or socioeconomic groups of the population. The highest rate of smoking was observed in men in the lowest wealth quintiles where almost one in every two men smoked (table 1). On the other hand, women in the low-income country group who were educated or wealthy were among the least likely to smoke. Less than 3% of women who had completed secondary or high school smoked, and only 3% of women in the highest wealth quintile smoked (table 1). This was strikingly different from the pattern seen in the middle-income country group where more than 10% of women smoked in all educational groups and in all wealth quintiles (table 1).

Unadjusted odds ratios for each of the demographic and socioeconomic factor studied can be found in Table S3. It is clear that prevalence of smoking varies across the 48 different countries in this study and that demographic and socioeconomic characteristics are often closely interlinked. For this reason we used multivariable logistic regression to calculate odds ratios adjusted for demographic and socioeconomic factors and for country of residence. In the middle-income country group, the odds of smoking increased with age until about 50 years old for both men and women after which they began to decrease. For example, men aged 40–49 were 1.2 (95% CI: 1.1–1.4) times more likely to smoke compared with those aged 18–29 after controlling for factors such as marital status, education, employment and wealth. Similarly, women of this age group were 1.2 (95% CI: 1.1–1.4) times more likely to smoke than their 18–29 year old counterpart (table 2). While in the middle-income country group, the odds of smoking in the oldest age groups for both men and women decreased to below the levels of the reference category (18–29 years), this pattern was markedly different for women in the low-income country group, where the adjusted odds ratios were highest in the oldest age groups. Women ages 50–59 were over 6 times more likely to smoke compared with women ages 18–29 (table 2). The pattern for men in the low-income country group was more similar to the middle-income country group except the magnitude of the effect was higher and although there was a reduction after age 50 years, the odds of smoking was never smaller than in the reference category (table 2).

**Table 2:** Multivariable analysis of the odds of current smoking by sex and country income group according to individual demographic and socioeconomic factors (data from the 2002–04 World Health Surveys of 48 low- or middle-income countries)*.

	Middle income**						Low income**					
	Male			Female			Male			Female		
	Adjusted odds ratio	(95%CI	)	Adjusted odds ratio	(95%CI	)	Adjusted odds ratio	(95%CI	)	Adjusted odds ratio	(95%CI	)
**Age**												
18–29	1	-		1	-		1	-		1	-	
30–39	1.16	(1.03–	1.29)	1.24	(1.08	–1.42)	1.64	(1.43	–1.89)	2.38	(1.84	–3.08)
40–49	1.21	(1.07–	1.36)	1.23	(1.05	–1.44)	1.89	(1.62	–2.21)	4.00	(2.73	–5.85)
50–59	0.96	(0.84–	1.10)	0.87	(0.73	–1.03)	1.68	(1.42	–2.00)	6.02	(4.21	–8.61)
60–69	0.73	(0.61–	0.87)	0.52	(0.41	–0.65)	1.48	(1.21	–1.81)	5.31	(3.65	–7.71)
70+	0.33	(0.27–	0.42)	0.26	(0.19	–0.34)	1.30	(1.00	–1.69)	6.14	(4.09	–9.22)
**Marital Status**												
Never married	1	-		1	-		1	-		1	-	
Married/cohabiting	1.10	(0.98–	1.24)	0.85	(0.74	–0.98)	1.29	(1.12	–1.49)	1.18	(0.78	–1.77)
Divorced/separated/widowed	1.79	(1.46–	2.19)	1.27	(1.06	–1.52)	1.59	(1.20	–2.10)	1.13	(0.72	–1.79)
**Education**												
No education	2.81	(2.23–	3.55)	3.21	(2.34	–4.40)	2.54	(2.01	–3.23)	3.77	(1.96	–7.26)
Less than primary	2.02	(1.64–	2.48)	2.46	(1.92	–3.16)	2.24	(1.72	–2.92)	3.08	(1.63	–5.82)
Primary completed	1.88	(1.60–	2.21)	1.64	(1.32	–2.05)	1.85	(1.45	–2.36)	2.43	(1.29	–4.58)
Secondary/high school completed	1.39	(1.21–	1.59)	1.06	(0.88	–1.27)	1.67	(1.32	–2.11)	2.07	(1.02	–4.20)
College completed/higher	1	-		1	-		1	-		1	-	
**Employment**												
Not working for pay	1	-		1	-		1	-		1	-	
Employed	1.13	(1.02–	1.25)	1.12	(1.01	–1.25)	1.53	(1.33	–1.75)	1.16	(0.89	–1.51)
**Main economic provider of household**												
No	1	-		1	-		1	-		1	-	
Yes	1.04	(0.94–	1.16)	1.15	(1.01	–1.31)	1.11	(0.97	–1.26)	1.19	(0.95	–1.48)
**Wealth**												
Quintile 1	1.36	(1.19–	1.56)	0.90	(0.73	–1.10)	1.67	(1.37	–2.04)	2.10	(1.30	–3.39)
Quintile 2	1.14	(1.00–	1.30)	0.83	(0.70	–0.99)	1.51	(1.26	–1.81)	1.68	(1.21	–2.33)
Quintile 3	1.14	(1.02–	1.28)	0.88	(0.75	–1.04)	1.30	(1.10	–1.53)	1.76	(1.23	–2.53)
Quintile 4	1.07	(0.96–	1.20)	0.96	(0.81	–1.12)	1.10	(0.94	–1.28)	1.33	(0.96	–1.83)
Quintile 5	1	-		1	-		1	-		1	-	
**Place of residence**												
Rural	1	-		1	-		1	-		1	-	
Urban	1.19	(1.08–	1.32)	1.53	(1.32	–1.77)	1.10	(0.96	–1.27)	0.80	(0.61	–1.05)

*Multivariable models included all variables listed in the table, and country of residence.

**World Development Report 2005.

Generally, divorced, separated or widowed individuals were more likely to smoke than married or cohabiting individuals and those who had never been married (table 2). This pattern was observed in all but women in the low-income country group where the differences in marital status groups were not significant. The protective effect of education on smoking was apparent in men and in women, in both middle-income and the low-income country group. Individuals with no education were generally about 3 times more likely to smoke than those with a college education on higher (table 2).

Smoking also appeared to be more prevalent in those employed compared with those not working for pay, and this was significant after controlling for age, education and wealth in all settings except women of the low-income country group. Furthermore, women of the middle-income country group were more likely to smoke if they were the main economic provider of the household (table 2). The effect of wealth was not equal in men and women from the middle-income or the low-income country groups. Examining the pattern in men, we found that the poorest men were more likely to smoke even after controlling for important factors such as age or education. This was apparent in both the low- and the middle-income country groups. However, the effect of wealth on the likelihood of women smoking was apparently quite different in the middle-income country group compared with the low-income country group. For example, women in the poorest quintile were twice as likely to smoke compared with women in the highest wealth quintile if they lived in a low income country. Nevertheless, if they lived in a middle income country, all wealth groups had similar rates of smoking and the adjusted odds ratios actually showed a decreased likely hood of smoking in poorer quintiles although this did not reach statistical significance except for quintile 2 (table 2). Finally, in the middle-income country group urban populations were more likely to smoke, while in the low-income country group there was no effect.

## Discussion

This study used pooled data from 48 countries taking part in the World Health Surveys to analyse the demographic and socioeconomic determinants of current smoking in men and in women living in low- or middle-income countries. We show that smoking levels are not equal in all demographic and socioeconomic groups of the population, and that the distribution of smoking among the population is not always the same in low- and middle-income countries. In our study, some factors were fairly stable across the four datasets studied: for example, individuals were more likely to smoke if they had little or no education, regardless of if they were male or female, or lived in a low or a middle income country. Nevertheless, other factors, notably age and wealth, showed a differential effect on smoking by sex or country income group. While the proportion of smokers decreased after age 50 in the middle-income country group, it remained high in the older age groups in the low-income country group. Furthermore, we found that wealth was inversely associated with smoking in the low-income country group but to a lesser extent, or not at all, in the middle-income country group.

These findings suggest that tobacco control initiatives in low-income countries must really be targeted at all age groups. The large proportion of smokers in older age groups is worrisome because it suggests smoking cessation is uncommon in these populations and older persons are especially vulnerable to adverse health conditions associated with smoking due to comorbidities and weakened immune systems. That smoking was strongly associated with wealth in the low-income country group was not entirely unexpected as the association between smoking and poverty has been widely observed [15]–[17]. Nevertheless it was surprising that the effect was less pronounced or absent in the middle-income country group. Our finding suggests in these countries, education is a more important determinant of smoking than poverty and that preventative efforts should aim at reducing tobacco consumption in poorly educated groups.

In fact education was a key determinant of smoking after controlling for age and socioeconomic factors regardless of the country-income group. Historically, in developed nations the tobacco epidemic began in elite and highly educated, and then spread to lower socioeconomic groups [23]. This trend has been shown to be quite different in developing countries where the more educated have tended to avoid smoking initiation perhaps due to the accessibility of information regarding the health risks [18]. Disparities in health behaviours are difficult to explain due to complex and competing, underlying factors. Individuals in the lowest education group may differ from individuals in the highest education group in terms other than education, such as socio-cultural characteristics, family background or current living arrangement. For example, one study concluded that a substantial portion of educational disparities seen in smoking rates can be attributable to factors shared by siblings that contribute to shortened educational careers [24].

It may seem surprising that employment was only marginally associated with smoking, but this may reflect the categorization used in this study. For example, our reference category “not working for pay” is made up of people seeking employment, as well as those who are not seeking employment because they are maintained by their spouse and work in the home. Additionally, we did not consider the type of employment and some studies have shown a higher prevalence of smoking people with working class jobs [15]. We also found that women in the middle-income country group who were the main economic provider of the household showed a moderate but significant increased risk of smoking. Personal economic freedom to use ones earnings to purchase cigarettes would be the most obvious reasoning behind this association. However, it is also possible, at least in middle-income countries, that the higher odds of smoking in women who were the main economic provider may be linked to the idea that tobacco smoking in women represents independence and modernity. This gender perspective may tell us why a similar effect was not seen in men in these middle-income countries. Finally, we found that residence in urban areas was related to increased likelihood of smoking in the middle-income country group. Similarly, studies in high-income countries have shown that individuals living in metropolitan areas are more likely to be current smokers than rural inhabitants [25]. The lack of an effect found in the low-income country group may reflect that the urban areas themselves are less developed than in middle-income countries.

Overall, whether or not a person chooses to smoke may be determined by many factors, such as socioeconomic context, working conditions, where one lives, availability of tobacco products, whether one has peers or family members who smoke, or even the level of cultural acceptance in smoking [26]. For example, women in Southeast Asia traditionally have a very low level of smoking and in this area it is not a social norm for women to smoke [6]. Furthermore, low education smokers are more likely to live with other smokers and less likely to seek assistance to quit smoking [27].

In this study we used pooled data from 27 middle-income countries and 21 low-income countries. It is clear that the distribution of smoking within each country in the pooled datasets may not be identical and this variation could influence our effect estimates. We did not explore the interaction effects of our study's independent variables within each of the countries. It should be noted, however, that we did include a country variable in our multivariate model in order to control for any potential confounding effect related to the individual countries. Our focus is to provide estimates of how smoking is distributed in the low-income and the middle-income country groups and what factors influence the variations in patterns. It is these variations across settings that will provide the nuanced evidence for policy and it is in these settings that decision making is made difficult by the lack of valid and appropriately detailed information.

It should be noted that we defined smoking as current smokers, which included both daily and non-daily smokers. Some studies suggest that non-daily smokers have distinct socio-demographic characteristics [28] and thus their inclusion could have influenced our estimations. However, when we restricted the analysis to daily smokers the results were very similar. We included non-daily smokers to be comparable with other studies and because their exposure to tobacco smoke still gives them an increased risk of the negative health consequences associated with smoking. However, one limitation of this study is that we did not consider the frequency and intensity of smoking.

Furthermore, we mainly considered individual level variables. We did not evaluate potential contextual effects such as neighbourhood, although living in the most disadvantaged areas has been associated with higher odds of smoking even after controlling for individual level socioeconomic factors [29]. That being said, we did find an association between current smoking and residence in urban areas in the middle-income country group. This finding is in line with the idea that ones living environment may play an important role in whether or not a person chooses to smoke. Finally, we limited our analyses to the socioeconomic conditions of the respondent at the time of survey without considering early life conditions. One study has shown that the socioeconomic conditions an individual experiences throughout his/her life course may accumulate to produce increased rates of smoking uptake and reduced rates of cessation [30].

It is possible that a selection bias may have occurred in the sampling process especially in countries with lower response rate, although we are not aware of evidence to suggest that this had occurred. The main reason for household non-response was inability to locate the selected households or the households refusing to participate even before a roster could be obtained. Given that these factors are unlikely to be associated with the outcome of interest for this analysis (tobacco smoking) we do not believe non-response will have influences the overall results.

Health levels vary across different groups of the population and such differences can be determined socially, demographically, economically or geographically. When differences are preventable or reversible, they represent health inequity which is considered a social injustice [31]–[33]. Differential exposure to known risk factors, such as tobacco smoke, is clearly linked to inequity as the burden of disease attributed to smoking is unfairly affecting the less educated, less wealthy parts of society. Given that effective tobacco control initiatives do exist, this excess morbidity and mortality is reducible. Unless comprehensive tobacco prevention measures are able to reach all areas of society the gaps will only continue to grow. Here we provide composite estimations of the distribution of tobacco smoking in low- and middle-income countries. The findings will be informative for policy makers and other decision makers, allowing them to tailor future policies, and target the most vulnerable populations.

## Supporting Information

Table S1 **Study population (final unweighted sample count) by sex and country, World Health Survey, 2002–2004.**
(DOC)

Table S2 **Weighted crude prevalence of current smoking by sex and country (data from the 2002–04 World Health Surveys of 48 low-or middle-income countries).**
(DOC)

Table S3 **Bivariable analysis of the odds of current smoking by sex and country income group according to individual demographic and socioeconomic factors (data from the 2002–2004 World Health Surveys of 48 low- or middle-income countries).**
(DOC)

List S1 **List of in-country collaborating partners.**
(XLS)
