# Supplementary material for: Social Determinants of Smoking in Low- and Middle-Income Countries: Results from the World Health Survey
Source: PLoS One. 2011 May 31;6(5):e20331. doi: 10.1371/journal.pone.0020331 (PMC3105024; doi:10.1371/journal.pone.0020331)
Supplement: Table S2 — Weighted crude prevalence of current smoking by sex and country (data from the 2002–04 World Health Surveys of 48 low-or middle-income countries). (DOC) [file pone.0020331.s002.doc]

**Table S2** Weighted crude prevalence of current smoking by sex and country (Data from the 2002-04 World Health Surveys of 48 low-or middle-income countries)

| **Middle income*** | | | | | | | **Low income*** | | | | | | |
| --- | --- | --- | --- | --- | --- | --- | --- | --- | --- | --- | --- | --- | --- |
| **Country** | **Males** | | | **Female** | | | **Country** | **Males** | | | **Female** | | |
|  | Prevalence (%) | (95%CI) | | Prevalence (%) | (95%CI) | |  | Prevalence (%) | (95%CI) | | Prevalence (%) | (95%CI) | |
| Bosnia and Herzegovina | 54.2 | (47.4 - | 60.9) | 34.0 | (24.3 - | 43.8) | Bangladesh | 56.1 | (53.3 - | 58.9) | 6.5 | (5.0 - | 8.0) |
| Brazil | 26.9 | (24.7 - | 29.2) | 17.8 | (16.0 - | 19.6) | Burkina Faso | 24.3 | (21.1 - | 27.5) | 11.5 | (8.4 - | 14.7) |
| China | 57.5 | (53.0 - | 62.0) | 3.4 | (2.3 - | 4.6) | Chad | 18.3 | (14.6 - | 22.0) | 3.3 | (1.4 - | 5.2) |
| Croatia | 30.6 | (25.4 - | 35.7) | 23.5 | (19.1 - | 27.9) | Comoros | 35.6 | (29.5 - | 41.7) | 22.2 | (14.7 - | 29.6) |
| Czech Republic | 37.1 | (29.5 - | 44.7) | 25.5 | (19.4 - | 31.5) | Congo | 17.3 | (12.6 - | 21.9) | 1.9 | (0.6 - | 3.1) |
| Dominican Republic | 17.3 | (14.9 - | 19.7) | 12.5 | (10.5 - | 14.4) | Cote d'Ivoire | 20.9 | (18.2 - | 23.7) | 2.9 | (1.8 - | 3.9) |
| Ecuador | 28.7 | (24.9 - | 32.5) | 7.1 | (5.3 - | 8.9) | Ethiopia | 7.4 | (5.3 - | 9.4) | 0.5 | (0.2 - | 0.9) |
| Estonia | 56.9 | (52.5 - | 61.4) | 25.1 | (22.0 - | 28.3) | Ghana | 10.6 | (8.7 - | 12.4) | 1.3 | (0.7 - | 1.8) |
| Georgia | 60.9 | (57.2 - | 64.5) | 6.4 | (3.8 - | 8.9) | India | 35.3 | (32.3 - | 38.4) | 7.6 | (5.4 - | 9.7) |
| Hungary | 43.7 | (37.7 - | 49.7) | 39.5 | (33.6 - | 45.4) | Kenya | 26.9 | (22.1 - | 31.7) | 2.0 | (0.9 - | 3.1) |
| Kazakhstan | 52.0 | (47.9 - | 56.1) | 9.6 | (6.0 - | 13.2) | Lao People's  Democratic Republic | 63.4 | (60.4 - | 66.5) | 13.0 | (10.2 - | 15.7) |
| Latvia | 64.7 | (58.4 - | 71.0) | 24.0 | (19.9 - | 28.1) | Malawi | 25.6 | (22.3 - | 28.8) | 5.7 | (4.0 - | 7.5) |
| Malaysia | 53.7 | (51.1 - | 56.2) | 2.6 | (1.8 - | 3.4) | Mali | 25.7 | (23.0 - | 28.3) | 3.0 | (1.7 - | 4.2) |
| Mauritius | 42.9 | (39.9 - | 45.9) | 2.8 | (1.7 - | 3.8) | Mauritania | 31.2 | (26.9 - | 35.5) | 5.0 | (3.3 - | 6.7) |
| Mexico | 36.2 | (34.7 - | 37.7) | 15.2 | (13.9 - | 16.4) | Myanmar | 47.5 | (44.3 - | 50.8) | 12.4 | (10.2 - | 14.6) |
| Morocco | 32.1 | (27.7 - | 36.5) | 0.2 | (0.0 - | 0.4) | Nepal | 33.5 | (31.0 - | 35.9) | 19.5 | (17.6 - | 21.3) |
| Namibia | 29.0 | (25.2 - | 32.8) | 12.4 | (10.0 - | 14.7) | Pakistan | 33.1 | (30.2 - | 35.9) | 6.4 | (5.1 - | 7.8) |
| Paraguay | 41.5 | (38.9 - | 44.1) | 13.3 | (11.8 - | 14.8) | Senegal | 25.4 | (21.3 - | 29.4) | 1.7 | (0.6 - | 2.9) |
| Philippines | 57.8 | (55.6 - | 60.0) | 12.5 | (11.2 - | 13.8) | Viet Nam | 51.4 | (46.0 - | 56.9) | 2.5 | (1.5 - | 3.5) |
| Russian Federation | 57.2 | (51.9 - | 62.5) | 11.2 | (8.9 - | 13.6) | Zambia | 23.8 | (21.4 - | 26.2) | 5.9 | (4.4 - | 7.5) |
| Slovakia | 41.6 | (31.9 - | 51.3) | 24.2 | (18.3 - | 30.1) | Zimbabwe | 26.3 | (23.2 - | 29.4) | 3.1 | (2.1 - | 4.1) |
| South Africa | 38.7 | (34.5 - | 42.9) | 12.5 | (9.9 - | 15.1) |  |  |  |  |  |  |  |
| Sri Lanka | 40.2 | (37.3 - | 43.2) | 3.0 | (1.9 - | 4.1) |  |  |  |  |  |  |  |
| Swaziland | 15.4 | (11.4 - | 19.4) | 3.2 | (1.5 - | 4.9) |  |  |  |  |  |  |  |
| Tunisia | 53.5 | (50.8 - | 56.3) | 2.2 | (1.6 - | 2.9) |  |  |  |  |  |  |  |
| Ukraine | 54.4 | (49.7 - | 59.1) | 10.7 | (8.4 - | 13.1) |  |  |  |  |  |  |  |
| Uruguay | 39.0 | (36.7 - | 41.3) | 28.8 | (25.1 - | 32.5) |  |  |  |  |  |  |  |

* World Development Report 2005
