# Supplementary material for: Social Determinants of Smoking in Low- and Middle-Income Countries: Results from the World Health Survey
Source: PLoS One. 2011 May 31;6(5):e20331. doi: 10.1371/journal.pone.0020331 (PMC3105024; doi:10.1371/journal.pone.0020331)
Supplement: Table S3 — Bivariable analysis of the odds of current smoking by sex and country income group according to individual demographic and socioeconomic factors (data from the 2002–2004 World Health Surveys of 48 low- or middle-income countries). (DOC) [file pone.0020331.s003.doc]

**Table S3** Bivariable analysis of the odds of current smoking by sex and country income group according to individual demographic and socioeconomic factors (Data from the 2002-04 World Health Surveys of 48 low- or middle-income countries)

|  | **Middle income*** | | | | | | |  | **Low income*** | | | | | | |
| --- | --- | --- | --- | --- | --- | --- | --- | --- | --- | --- | --- | --- | --- | --- | --- |
|  | **Male** | | |  | **Female** | | |  | **Male** | | |  | **Female** | | |
|  | Unadjusted odds ratio | (95%CI | ) |  | Unadjusted odds ratio | (95%CI | ) |  | Unadjusted odds ratio | (95%CI | ) |  | Unadjusted odds ratio | (95%CI | ) |
| **Age** |  |  |  |  |  |  |  |  |  |  |  |  |  |  |  |
| 18-29 | 1 | - |  |  | 1 | - |  |  | 1 | - |  |  | 1 | - |  |
| 30-39 | 1.31 | (1.19 - | 1.45) |  | 1.30 | (1.14 - | 1.47) |  | 2.09 | (1.87 - | 2.34) |  | 2.82 | (2.19 - | 3.64) |
| 40-49 | 1.52 | (1.38 - | 1.68) |  | 1.45 | (1.26 - | 1.67) |  | 2.64 | (2.34 - | 2.99) |  | 4.81 | (3.46 - | 6.69) |
| 50-59 | 1.24 | (1.11 - | 1.39) |  | 1.19 | (1.02 - | 1.39) |  | 2.22 | (1.95 - | 2.53) |  | 7.43 | (5.46 - | 10.11) |
| 60-69 | 1.09 | (0.95 - | 1.24) |  | 0.80 | (0.66 - | 0.97) |  | 1.97 | (1.67 - | 2.31) |  | 6.70 | (4.87 - | 9.21) |
| 70+ | 0.64 | (0.54 - | 0.75) |  | 0.48 | (0.38 - | 0.61) |  | 1.76 | (1.41 - | 2.19) |  | 7.65 | (5.30 - | 11.04) |
|  |  |  |  |  |  |  |  |  |  |  |  |  |  |  |  |
| **Marital Status** |  |  |  |  |  |  |  |  |  |  |  |  |  |  |  |
| Never married | 1 | - |  |  | 1 | - |  |  | 1 | - |  |  | 1 | - |  |
| Married/cohabiting | 1.27 | (1.17 - | 1.38) |  | 0.94 | (0.83 - | 1.07) |  | 2.29 | (2.07 - | 2.54) |  | 3.43 | (2.34 - | 5.04) |
| Divorced/separated | 1.80 | (1.51 - | 2.15) |  | 1.13 | (0.97 - | 1.32) |  | 2.24 | (1.78 - | 2.82) |  | 5.69 | (3.83 - | 8.45) |
|  |  |  |  |  |  |  |  |  |  |  |  |  |  |  |  |
| **Education** |  |  |  |  |  |  |  |  |  |  |  |  |  |  |  |
| No education | 1.01 | (0.84 - | 1.22) |  | 0.95 | (0.72 - | 1.24) |  | 2.82 | (2.19 - | 3.63) |  | 11.06 | (6.31 - | 19.36) |
| Less than primary | 0.98 | (0.82 - | 1.17) |  | 1.63 | (1.30 - | 2.04) |  | 2.77 | (2.10 - | 3.64) |  | 7.28 | (4.08 - | 12.99) |
| Primary completed | 1.00 | (0.86 - | 1.16) |  | 1.22 | (1.00 - | 1.50) |  | 2.00 | (1.56 - | 2.55) |  | 3.87 | (2.13 - | 7.03) |
| Secondary/high school completed | 0.92 | (0.81 - | 1.05) |  | 1.05 | (0.89 - | 1.25) |  | 1.60 | (1.25 - | 2.06) |  | 2.31 | (1.10 - | 4.86) |
| College completed/higher | 1 | - |  |  | 1 | - |  |  | 1 | - |  |  | 1 | - |  |
|  |  |  |  |  |  |  |  |  |  |  |  |  |  |  |  |
| **Employment** |  |  |  |  |  |  |  |  |  |  |  |  |  |  |  |
| Not working for pay | 1 | - |  |  | 1 | - |  |  | 1 | - |  |  | 1 | - |  |
| Employed | 1.26 | (1.16 - | 1.37) |  | 1.39 | (1.26 - | 1.54) |  | 2.26 | (2.00 - | 2.55) |  | 1.06 | (0.88 - | 1.28) |
|  |  |  |  |  |  |  |  |  |  |  |  |  |  |  |  |
| **Main economic provider of household** |  |  |  |  |  |  |  |  |  |  |  |  |  |  |  |
| No | 1 | - |  |  | 1 | - |  |  | 1 | - |  |  | 1 | - |  |
| Yes | 1.21 | (1.12 - | 1.31) |  | 1.09 | (0.98 - | 1.22) |  | 1.84 | (1.68 - | 2.01) |  | 1.31 | (1.09 - | 1.58) |
|  |  |  |  |  |  |  |  |  |  |  |  |  |  |  |  |
| **Wealth** |  |  |  |  |  |  |  |  |  |  |  |  |  |  |  |
| Quintile 1 | 1.52 | (1.34 - | 1.71) |  | 0.92 | (0.77 - | 1.10) |  | 2.29 | (1.94 - | 2.69) |  | 3.20 | (2.29 - | 4.48) |
| Quintile 2 | 1.27 | (1.13 - | 1.43) |  | 0.88 | (0.75 - | 1.04) |  | 1.92 | (1.65 - | 2.23) |  | 2.52 | (1.91 - | 3.33) |
| Quintile 3 | 1.23 | (1.10 - | 1.38) |  | 0.93 | (0.79 - | 1.09) |  | 1.52 | (1.32 - | 1.76) |  | 2.41 | (1.81 - | 3.21) |
| Quintile 4 | 1.15 | (1.02 - | 1.29) |  | 0.98 | (0.84 - | 1.15) |  | 1.21 | (1.06 - | 1.39) |  | 1.69 | (1.24 - | 2.30) |
| Quintile 5 | 1 | - |  |  | 1 | - |  |  | 1 | - |  |  | 1 | - |  |
|  |  |  |  |  |  |  |  |  |  |  |  |  |  |  |  |
| **Urbanization** |  |  |  |  |  |  |  |  |  |  |  |  |  |  |  |
| Rural | 1 | - |  |  | 1 | - |  |  | 1 | - |  |  | 1 | - |  |
| Urban | 0.88 | (0.80 - | 0.97) |  | 1.64 | (1.43 - | 1.88) |  | 0.81 | (0.71 - | 0.93) |  | 0.52 | (0.41 - | 0.67) |

* World Development Report 2005
